# Labeling of Disease-Modifying Therapies for Neurodegenerative Disorders

**DOI:** 10.3389/fmed.2019.00223

**Published:** 2019-10-17

**Authors:** Anne Vinther Morant, Vivien Jagalski, Henrik Tang Vestergaard

**Affiliations:** ^1^Regulatory Science & Advocacy, H. Lundbeck A/S, Copenhagen, Denmark; ^2^Regulatory Strategy, H. Lundbeck A/S, Copenhagen, Denmark

**Keywords:** disease modification, neurodegenerative disorders, label, indication, US Food and Drug Administration, European Medicines Agency, regulatory guidelines

## Abstract

Neurodegenerative disorders are characterized by progressive degeneration of nerve cells resulting in functional decline of cognition and/or movement. As the prevalence of many of these disorders increases with the aging global population, there is an urgent need for disease-modifying drugs that will halt or slow the progression of these devastating diseases. A summary of the scientific information needed to guide the safe and effective use of a drug is provided in the product label in which the indication section should clearly state the treatment concept, e.g., distinguish between symptomatic, preventive, and curative treatments. However, a review of the United States (US) and European Union (EU) product labels for disease-modifying multiple sclerosis (MS) drugs reveals that the indications are not aligned with the regulatory guidance on labeling. Indication claims such as “delay of accumulation of disability” and “slowing of disease progression” were previously accepted by the US Food and Drug Administration (FDA) and the European Medicines Agency (EMA); however, all recently approved MS drugs include no such specification of the treatment concept in the label indication sections despite similar clinical data packages supporting the approvals. Coincidently, the FDA and EMA therapeutic guidelines pertaining to development of drugs for treatment of neurodegenerative disorders have changed from providing recommendations for specific disease modification label claims to a more general focus on the clinical development approach. Our analysis of MS drug labels could imply that the FDA and EMA may be unlikely to accept disease modification-related indication claims for drugs to treat neurodegenerative disorders in general. We envision that a potential disease-modifying effect is more likely to be inferred from the label descriptions of the mechanism of action, clinical efficacy data and trial design, and target patient population. This poses a challenge for communication of the clinical benefit in a language that can be easily understood by patients and prescribers.

## Introduction

### Distinguishing Disease-Modifying From Symptomatic Treatments in Neurodegenerative Disorders

For drugs in development for treatment of neurodegenerative disorders, a distinction is often made between drugs that mediate their effect through targeting the underlying pathophysiology with the aim of obtaining an enduring clinical benefit (referred to as disease-modifying drugs) and those that relieve symptoms without targeting the underlying pathophysiology (referred to as symptomatic drugs). However, the conceptual definition of “disease modification” varies both within and between neurodegenerative disorders (1–3). In addition, the meaningfulness of distinguishing between disease-modifying and symptomatic treatments has been debated (4).

Examples of neurodegenerative disorders include Huntington's disease (HD), amyotrophic lateral sclerosis (ALS), frontotemporal dementia, Parkinson's disease (PD), and Alzheimer's disease (AD). To date, only symptomatic drugs have been approved for PD, for chorea associated with HD, and for the dementia stages of AD. Furthermore, no novel therapeutic drugs have been approved for treatment of AD for more than a decade. As the prevalence of AD and other neurodegenerative disorders is increasing dramatically with the globally aging population (5), there is a growing need for effective treatments that could delay or slow the progression of these devastating diseases. Numerous efforts are and have been underway to develop disease-modifying therapies that target the underlying pathophysiological changes in AD and to some extent other neurodegenerative disorders (6), but the complexity, duration and significant costs of clinical development in combination with the high attrition rates have unfortunately discouraged some sponsors from continuing the efforts to develop these urgently needed therapies.

The regulatory and clinical development paths for demonstrating a disease-modifying drug effect in neurodegenerative disorders are arguably significantly more complex compared to the development paths for symptomatic treatments (3, 7, 8) that per definition treat stages of the diseases where clinical symptoms are apparent. To ensure that such potential new treatment concepts are clearly explained to prescribers and patients, there is a particular interest in conceptually distinguishing disease-modifying treatments from symptomatic-only treatments in the product label as observed for certain drugs approved for the treatment of multiple sclerosis (MS) and rheumatoid arthritis (RA) (9). This is of course providing that sponsors—in spite of the complexity of the disease biology and clinical development—will be able to successfully provide adequate data to convince regulatory authorities that a meaningful and lasting change in the clinical course and the underlying pathophysiology of the disease has been demonstrated.

### The Product Label: Key Tool for Communicating Drug Characteristics to Patients and Prescribers

The US Prescribing Information (USPI) and EU Summary of Product Characteristics (SmPC; collectively referred to as the product label) are an integral part of the approval of a novel therapeutic drug. The product label provides a summary of the scientific information needed to guide the safe and effective use of the drug and is hence a key tool to inform prescribing decisions (10–12). Furthermore, the product label is *per se* the most important tool for communication of the key characteristics (indication, posology, safety, efficacy, and storage) to healthcare professionals (13, 14) and constitutes the main substantiation for promotional materials (15, 16).

To eliminate unnecessary risks and futile treatment, it is key to provide clear and adequate guidance to prescribers and patients. Therefore, it is important to ensure that the product label clearly describes the treatment concept and efficacy profile in a well-defined patient population. These two elements—the treatment concept and the drug effect—together with the target patient population are described in dedicated sections in the product label:

- The *Indications and Usage* (section 1 of the USPI) and *Therapeutic Indication* (section 4.1 of the SmPC) sections (collectively referred to as the indication section) describe the target population and disease or condition for which the drug is approved.
- The *Clinical Studies* (section 14 of the USPI) or *Pharmacodynamic Properties* (section 5.1 of the SmPC) sections (collectively referred to as the clinical efficacy section) outline the details of the clinical efficacy data supporting the indication(s).

The US and EU regulations (including regulatory guidance) specify that the indication(s) should be stated clearly and concisely to support prescribing decisions (10, 12, 17, 18). A description of the target population should also be included especially when restrictions to the population apply or when the drug is approved for specific subpopulations (10, 18).

Further outlining the treatment concept, the US *Indications and Usage* section must state whether the drug is indicated for the “*treatment, prevention, mitigation, cure, or diagnosis of a recognized disease or condition, or of a manifestation of a recognized disease or condition, or for the relief of symptoms associated with a recognized disease or condition*” (18). Similarly in the EU, the indication should distinguish “*between treatment (symptomatic, curative, or modifying the evolution or the progression of the disease), prevention (primary or secondary), and diagnostic*” (10).

In EU, as opposed to the US, the essential information from the SmPC is further translated into lay language in the Package Leaflet which is intended for the end-user, i.e., the patient and/or the caregiver (19). This provides an opportunity—and an obligation—to communicate to patients what the drug is and how it works, i.e., the treatment concept.

With future regulatory approvals of potentially disease-modifying treatments for neurodegenerative disorders in mind, the aim of this analysis was to explore the current regulatory trends in terms of the vocabulary used to describe drugs targeting the underlying pathophysiology of neurodegenerative disorders. We reviewed the terminology applied in regulatory therapeutic guidelines and product labels for disease-modifying drugs approved by the FDA and the EMA for the treatment of MS and other neurodegenerative disorders.

## Methods

For all analyses, data were extracted independently, reviewed and verified by at least two authors, and discrepancies were resolved by discussion and consensus.

### Disease Modification-Related Terminology

We defined “disease modification-related terminology” as any wording that directly (disease modification; disease-modifying) or indirectly (delay of disability; delay or slowing of disease progression; prevention; effect on/change of disease course) implies an enduring change in the pathophysiological processes that constitute the underlying cause of the disease.

### Regulatory Therapeutic Guidelines

FDA and EMA Committee for Medicinal Products for Human use (CHMP) therapeutic development guidelines and discussion papers pertaining to neurodegenerative disorders were identified from the respective agency websites (20, 21). The obsolete FDA guidances were sourced from the Cortellis Database (22) and www.researchgate.net.

Guidelines were analyzed for disease modification-related terminology using the search terms: Indication; claim; disease modif^*^; progression; prevent^*^; delay of ^*^; and disease course.

### Regulatory Precedence: Current Drug Labels

USPIs were retrieved from Drugs@FDA (23), and EU SmPCs were retrieved from the EMA European Public Assessment Reports (EPAR) website (24) or—for drugs not approved via the EMA Centralized Procedure—from the UK Electronic Medicines Compendium (25).

### Regulatory Precedence for Disease-Modifying Treatments: Multiple Sclerosis

Drugs approved for treatment of MS by both the FDA and EMA (Centralized Procedure) were identified from the respective agency websites as listed above. The initially approved indications were retrieved from the original SmPCs and USPIs obtained from the Cortellis Database (22), Drugs@FDA (23), and the DailyMed Label Archives^1^. Initial EU Package Leaflets were obtained from the Cortellis Database. Information on the pivotal clinical trials supporting FDA and EMA approval was retrieved from the FDA reviews (or—in a few cases when not publicly available—the USPI) and EPARs. The analysis focused on the study design including comparator (active or placebo) and the three main clinical outcome measures (relapse measures, disability progression, and magnetic resonance imaging [MRI] measures). Only products with a novel active ingredient were considered excluding generics, biosimilars, and drugs approved via informed consent applications (meaning the originator medicine's marketing authorization holder consented to the use of the originator's medicine's data for the application before the end of the data protection period). For EU, information on the indication originally proposed by the company were retrieved from the EPARs.

## Regulatory Therapeutic Development Guideline Terminology

### Terminology Applied in Current Regulatory Guidelines for Development of Drugs to Treat Neurodegenerative Disorders

A systematic search of available regulatory therapeutic development guidelines showed that the EMA (CHMP) generally applies “disease modification” and related terminology in regulatory guidelines for neurodegenerative disorders (Table 1). The FDA, in contrast, does not explicitly apply “disease modification” terminology in their current regulatory guidelines. Nevertheless, the FDA applies related terminology (“*persistent effect on disease course*”) in the current AD draft guidance (7) (Table 1).

**Table 1:** Current regulatory development guidelines for neurodegenerative disorders.

**Therapeutic area**	**FDA terminology**	**EMA (CHMP) terminology**	**References**
Amyotrophic lateral sclerosis	No terminology related to disease modification	• Disease modification • Prevention, delay or modification of disease progression	(26) (27)
Alzheimer's disease	• Persistent effect on disease course • Direct effect on the underlying disease pathophysiology	• Disease modification • Persistent delay in the underlying neuropathological process • Delay of clinical decline	(7) (3)
Duchenne muscular dystrophy	No terminology related to disease modification (mention of treatment directed at the underlying disease pathology)	• Disease modification • Delay in disease progression	(28) (29)
Multiple sclerosis	N/A—no FDA guidance available	• Disease modification • Modification of disease progression • Delay of accumulation of disability	(2)
Parkinson's disease	N/A—no FDA guidance available	• Disease modification • Delay in disease progression	(8)

*Disease modification and related terminology applied in current FDA and CHMP development guidelines pertaining to neurodegenerative disorders*.

### Regulatory Alzheimer's Disease Development Guidelines: Terminology Changes Over Time

**Figure 1:**
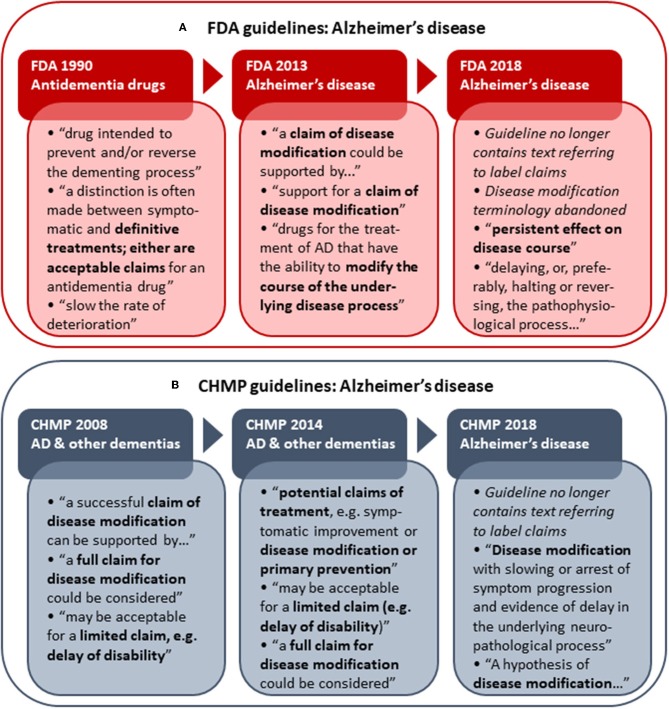
Historical development in FDA **(A)** and CHMP **(B)** Alzheimer's disease (AD) development guidelines with respect to disease modification terminology and reference to actual label claims. Examples of terminology applied in the respective guidelines are shown. The first CHMP guideline for development of drugs to treat AD and other dementias was adopted by the CHMP in 2008 (30). A CHMP discussion paper on AD and other dementias was published in 2014 (31) as part of the revision of the guideline, which was finally adopted by the CHMP in 2018 (3). The FDA released its first draft guidance pertaining to AD (antidementia drugs) in 1990 (32). The FDA draft guidance for industry pertaining to the pre-dementia stages of AD was first published in 2013 (33) and replaced with an updated version in 2018 (7).

As several versions of guidelines for development of drugs to treat AD have been published from both the FDA and EMA, these provide an opportunity to examine the development of disease modification-related terminology applied in regulatory guidelines over time. The analysis of obsolete, draft and current FDA and CHMP guidelines (including a CHMP discussion paper) for development of drugs for the treatment of AD showed that the CHMP retains “disease modification” terminology from 2008 (30) through 2014 (31) and 2018 (3), while the FDA has abandoned this terminology in the recently updated 2018 draft guidance (7) (Figure 1). The FDA guidance now refers to a “*persistent effect on disease course*” instead of a disease-modifying effect. In addition, whereas the guidelines from both agencies previously provided recommendations on how to obtain a specific label claim for e.g., disease modification (30, 31, 33), the guidelines from 2018 provide recommendations relating to treatment goals only, i.e., without guidance on how to obtain specific label claims (3, 7).

## Regulatory Precedence: Label Indications for Drugs Approved for Treatment of Neurodegenerative Disorders

### Label Indications for Symptomatic Therapies for Neurodegenerative Disorders

To illustrate the current regulatory environment in terms of labeling practice for drugs for symptomatic treatment of neurodegenerative disorders, examples of label indication terminology are outlined below.

To date, only symptomatic drugs have been approved for treatment of AD, PD, and HD. For the AD drugs, the USPI indication sections merely state “treatment of dementia of the Alzheimer's type” with specification of the specific AD stages (34–37), while the EU SmPC indication sections for the cholinesterase inhibitors explicitly state “symptomatic treatment of Alzheimer's dementia” (38–40). In contrast, the EU SmPC indication for memantine does not specify “symptomatic” and is indicated for “treatment of AD” (41). According to the current regulatory guidelines on product labeling (10, 18), the treatment concept (in this case “symptomatic”) must be stated in the indication section. However, although “symptomatic” is not specified in the indication section of the USPIs for the cholinesterase inhibitors, any effect beyond symptomatic relief is clearly dismissed in the USPI Clinical Trial sections as it is stated that there is no evidence that the drugs alter the course of the underlying dementing process.

Within PD, a range of symptomatic drugs with different mechanisms of action are approved in both US and EU. For most of these, both the USPI and SmPC indication sections simply state “treatment of (idiopathic) PD,” while only a few SmPCs explicitly state “for treatment of the signs and symptoms” or otherwise imply a symptomatic effect (42–49).

In contrast, for the drugs approved for the treatment of patients with HD, the indication sections clearly specify that the drugs are for treatment of chorea associated with HD as opposed to treatment of HD *per se* (50–53).

### Label Indication Terminology for Disease-Modifying Therapies: Multiple Sclerosis

**Figure 2:**
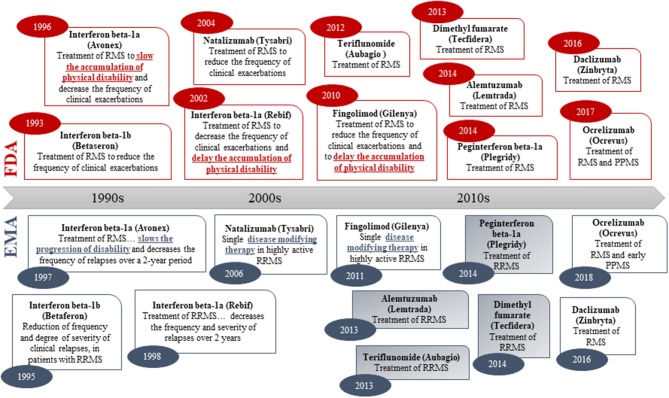
Original indications (abbreviated) of medicines approved for treatment of multiple sclerosis in both the US (FDA) and the EU (via the EMA Centralized Procedure). Numbers in oval blue and read shapes signify approval year. Blue/gray shading signifies that the sponsor applied for an indication with disease modification-related terminology in the EU. Daclizumab was withdrawn in the EU in 2018.

To examine labeling practices for disease-modifying therapies, a systematic analysis of labels for drugs to treat MS was performed, as—in contrast to AD, PD, and HD—several disease-modifying drugs have been approved for the treatment of MS (54). Although different levels of disease biology understanding (including link between clinical and biomarker outcomes) may influence the regulatory assessment, the regulatory precedence from MS provides an opportunity for studying the label claims of approved disease-modifying drugs for treatment of a disease that also has an important neurodegenerative component. The chronological review of the indication wording in the product labels for approved MS drugs shows that both the FDA and EMA have previously accepted disease modification-related terminology in the indication section (i.e., “delaying the accumulation of physical disability” in US vs. “slowing progression of disability” or “disease modifying therapy” in EU; Figure 2). However, none of the MS drugs approved since 2010 (US) and 2011 (EU) have wording related to disease modification or delay of disability in the indication section. Hence, there is a clear regulatory trend toward a simpler wording of the indication claim (i.e., “treatment of multiple sclerosis”) in both the US and EU (Figure 2).

**Figure 3:**
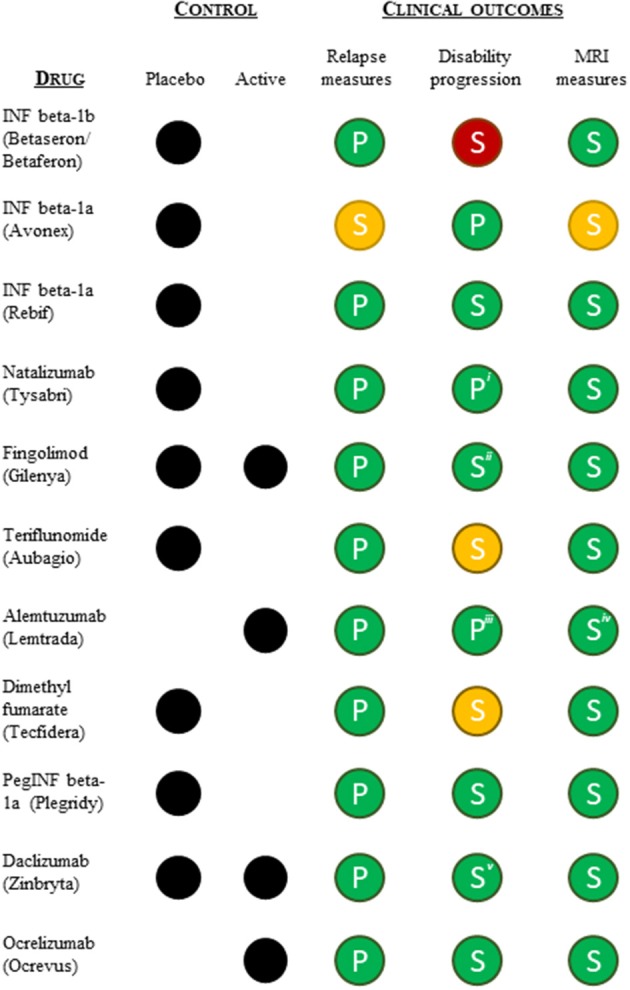
Summary overview of the pivotal clinical data (type of control group and clinical outcomes) supporting FDA and EMA approvals of the relapsing MS indication. Relapse measures included one or more of the following efficacy endpoints: annual relapse/exacerbation rate or proportion of patients free from relapses/exacerbation at a given time. Disability progression measures included an evaluation of progression of disability using the Kurtzke's Expanded Disability Status Scale (EDSS). MRI measures included MRI derived parameters used for monitoring CNS lesions. Green, statistically significant positive outcome(s); yellow, ambiguous positive/negative outcomes; red, statistically insignificant outcome(s); P, (co-)primary outcome measure; S, secondary outcome measure. ^*i*^FDA accelerated approval based on 1-year data, meaning the co-primary endpoint of 2-year disability progression was not included in support of the FDA approval. ^*ii*^Only the placebo-controlled pivotal trial was positive on the disability progression measure. ^*iii*^Only one of the two active-controlled pivotal trials was positive on the disability progression measure. ^*iv*^One of the pivotal studies was clearly positive on all MRI measures, while the second study showed mixed results. ^*v*^The active-controlled pivotal trial showed mixed disability progression results.

To understand whether differences in the pivotal clinical data submitted in support of the approved indications could explain the shift toward lack of disease modification-related terminology in the indications, we reviewed the design and outcomes of the pivotal clinical trials supporting the initial approval (excluding the ocrelizumab PPMS indication). All pivotal trials were randomized and controlled (placebo or active) and had a measure of relapse as the primary (or co-primary) endpoint (except interferon beta-1a). All were positive on the relapse measure (Figure 3). All pivotal programs included measures of disability progression as (co-)primary or secondary measures, and all except interferon beta-1b (negative), dimethyl fumarate and teriflunomide (one positive and one negative or mixed results) were positive on these measures. Lastly, all pivotal trial programs included imaging (MRI) measures of disease burden as secondary endpoints, and all except interferon beta-1a were clearly positive on these measures as well (Figure 3). In conclusion, we found no apparent differences in the pivotal trial designs or outcomes that could explain the shift in label indication terminology.

Information on the originally sponsor-proposed indication is fully disclosed in the EPARs. As such, our review showed that for four of the six drugs approved for MS after 2011 via the EMA Centralized Procedure, the sponsors originally applied for an indication with a disease modification-related indication claim (i.e., “delay/slow or reverse the accumulation/progression of physical disability,” and/or “disease-modifying therapy”) but these were rejected by the CHMP (Figure 2). The EPARs—while systematically referring to disease-modifying therapies—revealed that the CHMP in all four cases concluded that the claim was not sufficiently supported by the submitted data (55–58).

In Europe, the essentials of the SmPC are “translated” into lay language for the patient and/or caregivers in the Package Leaflet. We reviewed the initial Package Leaflet descriptions of “what the drug is” and “how the drug works” and compared to the same information in the SmPC indication sections to evaluate how the treatment concept is explained to the patients. Interestingly, the majority of the drugs that were approved after 2011 do include descriptions of a potentially disease-modifying effect in the Package Leaflet, even if no such effect is explicitly worded in the SmPCs (Table 2). The dynamics behind these discrepancies are not obvious, i.e., it is unclear whether the aim is to ensure consistency between products with similar indications and mode of actions rather than to ensure consistency between the Package Leaflet and the SmPC.

**Table 2:** EU Package Leaflet information for patients for multiple sclerosis disease-modifying drugs.

**Drug**	**SmPC indication**	**Information to patients on effects of the drug included in the initial Package Leaflet**
Interferon beta-1b (Betaferon)	• Reduction of frequency and degree of severity of clinical relapses in ambulatory patients with RRMS • Reduction in frequency and severity of clinical relapses and number of hospitalizations • Prolongation of the relapse-free interval • There is no evidence of an effect on the progression of the disease. • There is no evidence of an effect on disability	• Shown to modify the immune system response • Shown to reduce the frequency and severity of clinical relapses, to reduce the number of MS related hospitalizations and to prolong the relapse-free time • There is no evidence of an effect on the length of attacks, symptoms in between attacks, or the progression of the disease • There is no evidence of an effect on disability
Interferon beta-1a (Avonex)	• Treatment of ambulatory patients with RMS • Slows the progression of disability and decreases the frequency of relapses	• Shown to be useful in slowing the progression of the disease and reducing the number of flares
Interferon beta-1a (Rebif)	• Treatment of ambulatory patients with RRMS • Decreases the frequency and severity of relapses	• Shown to reduce the number and the severity of relapses and to increase the time between relapses.
Natalizumab (Tysabri)	• Single disease modifying therapy in highly active RRMS *plus definition of target patient groups*	• Stops the cells that cause inflammation from going into your brain. This reduces nerve damage caused by MS • Approximately halved the progression of the disabling effects of MS and also decreased the number of MS attacks by about two-thirds • Cannot repair the damage that has already been caused by MS • May still be working to prevent your MS becoming worse.
Fingolimod (Gilenya)	• Single disease modifying therapy in highly active RRMS *plus detailed definition of target patient groups*	• Does not cure MS, but it helps to reduce the number of relapses and to slow down the progression of physical disabilities due to MS • Helps to protect against attacks on the CNS by the immune system by reducing the ability of some white blood cells to move freely within the body and by stopping them from reaching the brain and spinal cord. This limits nerve damage caused by MS.
Teriflunomide (Aubagio)	• Treatment of patients with RRMS	• Helps to protect against attacks on the central nervous system by the immune system by limiting the increase of some white blood cells. This limits the inflammation that leads to nerve damage in MS.
Alemtuzumab (Lemtrada)	• Indicated for patients with RRMS	• Does not cure MS, but it can reduce the number of MS relapses • Can also help to slow down or reverse some of the signs and symptoms of MS • Patients treated with LEMTRADA had fewer relapses and were less likely to experience worsening of their disability compared to patients treated with a beta-interferon
Peginterferon beta-1a (Plegridy)	• Treatment of RRMS	• Seems to work by stopping the body's immune system from damaging your brain and spinal cord. This can help to reduce the number of relapses that you have and slow down the disabling effects of MS • Can help to prevent you from getting worse, although it will not cure MS
Dimethyl fumarate (Tecfidera)	• Treatment of RRMS	• Seems to work by stopping the body's defense system from damaging your brain and spinal cord. This may also help to delay future worsening of your MS
Daclizumab^*^ (Zinbryta)	• Treatment of RMS	• Works by stopping the body's immune system from damaging your brain and spinal cord. This can help to reduce the number of relapses that you have and slow down the disabling effects of MS • Can help to prevent you from getting worse, although it will not cure MS
Ocrelizumab (Ocrevus)	• Treatment of RMS • Treatment of early PPMS	• Targets and removes specific B cells. This reduces inflammation and attacks on the myelin sheath, reduces the chance of having a relapse, and slows the progression of your disease • In RMS, helps to significantly reduce the number of attacks (relapses) and significantly slow down the progression of the disease. Significantly increases the chance of a patient having no evidence of disease activity (brain lesions, relapses and worsening of disability) • In PPMS, helps to slow down the progression of the disease and reduce deterioration in walking speed

*Description of treatment concept in original EU SmPC indications and Package Leaflets (information on what the drug is and what it is used for). Green, Treatment concept implying a disease-modifying effect; Red, Disease-modifying effect explicitly dismissed; No color, No disease-modifying effect implied*.

* *Withdrawn in 2018*.

### Other Examples of Disease-Modifying Therapies: Rheumatoid Arthritis and Rare Neurodegenerative Disorders

**Figure 4:**
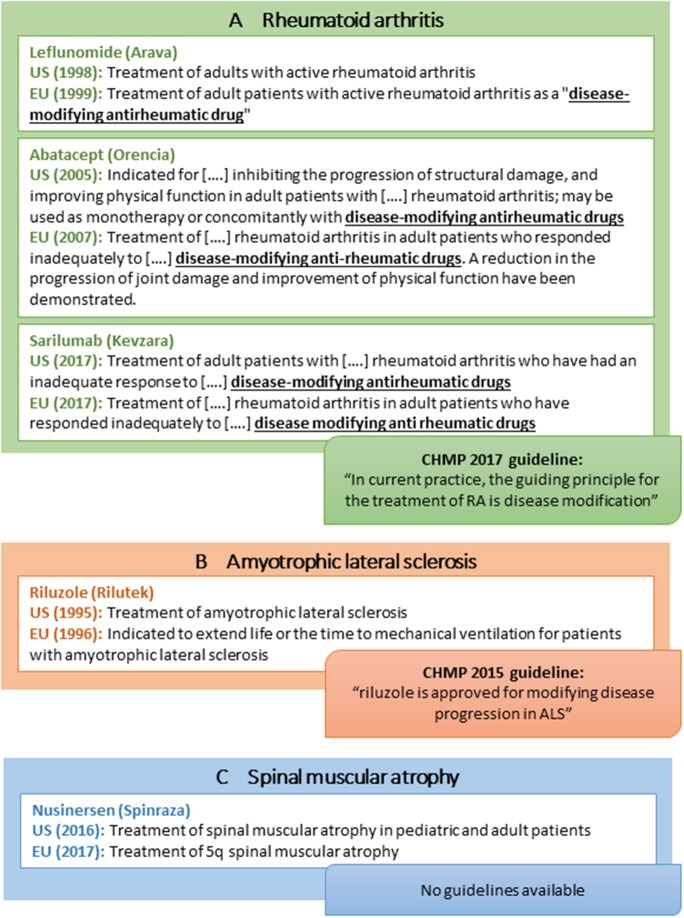
Selected examples of indications of disease-modifying drugs for rheumatoid arthritis **(A)**, amyotrophic lateral sclerosis **(B)**, spinal muscular atrophy **(C)**, and CHMP regulatory guideline use of disease modification-related terminology.

In addition to drugs for treatment of MS, learnings from rare neurodegenerative disorders and RA provide additional considerations for the acceptance of disease modification-related terminology (Figure 4):

- Within RA, one drug (leflunomide; Figure 4a) has an indication claim for disease modification in the EU SmPC only, although the claim is emphasized in quotation marks (Figure 4a). Notably, the indication sections of many FDA and EMA-approved RA drugs refer to “other disease-modifying anti-rheumatic drugs” although such wording is not included in the product labels for these other anti-rheumatic drugs. Likewise, the CHMP guideline refers to disease-modifying therapy as part of the current treatment landscape in RA (Figure 4a) (59).
- Within ALS, riluzole is approved for “Treatment of ALS” in the US but has a more elaborate indication in the EU (i.e., “…*extend life or the time to mechanical ventilation for patients with ALS*”; Figure 4b). Moreover, the CHMP ALS guideline from 2015 states that “*riluzole is approved for modifying disease progression in ALS*” (27). From a US perspective, no disease modification-related terminology is applied in the corresponding FDA guidance (Table 1) (26).
- Within spinal muscular atrophy (SMA), nusinersen was recently approved in both the US and the EU with no wording pertaining to disease modification or delay of progression included in the US and EU product label (Figure 4c) (60, 61). The FDA review does not discuss this issue (62), and the EPAR is speculative (e.g., “…*the initiation of treatment before the onset of clinical symptoms has the potential to delay or even prevent the progression of SMA*”) (63). Of note, the sponsor did not apply for a disease modification indication as the approved indication seems to be identical to the sponsor-proposed indication in both regions (62, 63).

## Disease Modification Terminology: Discussion of Regulatory Trends

### Regulatory Trend Toward Brief and General Indication Claims

The regulatory precedence points to a trend toward a higher level of conformity with brief indication claims specifying “treatment of” without further specification of the treatment concept or potential therapeutic effect of the treatment (i.e., slowing progression or delay of disability).

This trend seems not to be entirely in line with the current regulatory labeling guidance (10, 18) as detailed in the Introduction. Per the FDA and EMA guidance, the treatment concept should be clearly distinguished in the label (10, 18) and the information in the indication section should be “*useful and informative*” (18). We would argue that information on what the drug does and what this means to the patient—i.e., the treatment concept—would be both useful and informative to guide the optimal treatment for the individual patient.

Interestingly, Leber (Director of the FDA Division of Neuropharmacological Drug Products at the time) argued in 1997 that a distinction *must* be made between AD treatments that provide a symptomatic benefit and those that alter the course of dementia in order to avoid false or misleading label claims (64).

Regardless of the terminology used in the label, the main objective remains to provide a clinically meaningful treatment for the patients. This is supported by FDA and EMA regulators arguing that “*a small clinical effect that might be shown to be disease-modifying may in principle be less meaningful to the patient than another treatment resulting in a large symptomatic effect*” (65).

### Clarifying the Treatment Concept in Labels for Disease-Modifying Drugs

“Disease modification” has been described as “an inferential concept” that is based on clinical and biomarker observations from adequately designed clinical trials, as the changes in the brain cannot be directly measured or observed (1). This might explain the regulatory reluctance to accept direct label claims of “disease modification” as observed in the regulatory precedence analysis. In contrast, claims such as “slowing of progression” or “delay of [a given clinical milestone]” could potentially be substantiated by data from appropriately designed clinical studies and as such included in labeling as also suggested by Cummings (9). However, the review of MS drug labels suggests that such information might not be included in the indication section, but rather in the clinical efficacy section as a description of the observed clinical effect on the specific outcome measures without further translation into an explicitly phrased treatment concept.

However, even within and across the different regulatory documents (SmPCs, EPARs, and Package Leaflets) we observed a discrepancy in the communication and discussions of the disease-modifying treatment concept as exemplified by alemtuzumab. In this case, the sponsor applied for a disease modification-related indication (“…*indicated for adult patients with RRMS to decrease the frequency of relapses and slow or reverse accumulation of disability*”), which was rejected by the CHMP who argued in the EPAR that such a claim could be considered “…*misleading for the patient who could expect a healing of the disease*” (55). In the same EPAR, the CHMP interestingly endorsed including patients at early stages of the disease to “*allow for disease modification*” (55). Finally, the Package Leaflet lay language strongly indicates a disease-modifying effect by stating that alemtuzumab can “…*help to slow down or reverse some of the signs and symptoms of MS*” and that patients treated with alemtuzumab “…*were less likely to experience worsening of their disability*” compared to patients treated with the active comparator (Table 2).

For disease-modifying therapies for neurodegenerative diseases in general we suggest—based on the analysis of MS labels—that clarification of the treatment concept in terms of implying a potential disease-modifying effect might be most likely to result from the following label descriptions:

- mechanism of action (indication and section 12.1 of the USPI, and section 5.1 of the SmPC) describing the link between the drug target and the underlying pathophysiology,
- results of clinical studies (clinical efficacy section) designed to demonstrate a delay in disease progression or disability, and
- patient population (clinical efficacy section and most likely also the indication section)

For future potential AD disease-modifying drugs, the label description of the patient population could indirectly imply the treatment concept as the rationale for treating patients at an early (pre-dementia) disease stage where symptoms do not yet result in significant functional impairment would in most cases be slowing the progression rather than (or in addition to) symptomatic relief. This in contrast may not be the case for disorders where the disease-modifying drugs might target the same or overlapping patient populations as the symptomatic treatments.

In theory, the added value of disease-modifying therapies lies in the potential long-term effect, i.e., the slowing of progression of the disease, whereas the short-term effect may be more modest, especially in comparison to existing symptomatic therapies. The value assessment leading to health technology assessment (HTA) recommendations is exceedingly complex and varies widely across regions as exemplified by a recent analysis of HTA evaluations of MS disease-modifying therapies (66). Hence it may be difficult to single out any implication of the explicit regulatory endorsement of a disease-modifying effect embedded in a direct label claim. Even so, it is hard to imagine that an—at best—inferred disease-modifying effect should suffice as a basis for modeling of long-term effectiveness to convince HTA bodies and payers of the added value of future disease-modifying therapies. This would be an even bigger challenge within disorders where symptomatic therapies (including generics) are already available.

### Limitations of the Analysis

The analysis did not take into consideration any potential differences across the different therapeutic areas in terms of regulatory requirements, scientific understanding of disease biology, effect sizes demonstrated for the individual drugs, feasibility of conducting clinical trials, or availability of objective and sensitive clinical outcome measures and biomarkers that reliably detect pathophysiological changes. In the absence of approval of disease-modifying drugs for treatment of many neurodegenerative disorders, suggestions on how and if a disease-modifying treatment concept may or may not be accepted by regulators for inclusion in the label indication or clinical efficacy sections remain speculative.

Moreover, there are several factors—in addition to the design and results of the clinical trials—that influence the final indication terminology. The approved indication wording is a result of negotiations between the sponsor (who proposes an indication in their application for marketing authorization based on the submitted clinical data) and the regulatory authority (who evaluates whether the available data support the proposed claims).

## Actionable Recommendations and Conclusions

### Actionable Recommendations

Based on the results of our analysis, we propose the following recommendations listed below by region.

- **EU:**
  ° **Recommendation 1:** The EMA could consider ways of increasing transparency on how indication wordings are assessed in context of reviews of applications for marketing authorization. This could include the rationale for accepting or rejecting specific elements of the sponsor-proposed indication in the EPAR especially for drugs with a novel mode of action and/or drugs that differ from previously approved treatments within the same indication. This would be in line with other efforts aimed at ensuring a more transparent regulatory decision process. Specifically, a clear rationale for the decision would be desirable in cases where the final indication wording is not in line with prior approvals and/or regulatory guideline recommendations.
  ° **Recommendation 2:** The European Commission and the EMA could consider activities aimed at improving the consistency in product information terminology and description of treatment concepts. Focus should be to ensure the Package Leaflet reflects the description of the treatment concept in the SmPC both within and across therapeutic areas. Relevant stakeholders including patient organizations, health care professionals, and industry trade organizations should preferentially be consulted.

- **US:**
  ° **Recommendation 3:** The FDA could consider disclosing the sponsor-proposed indication (if possible for proprietary reasons) in the FDA review. Regardless, we encourage the FDA to implement a higher level of transparency as suggested for the EMA in Recommendation 1, i.e., providing the rationale for the regulatory decision to approve the final indication wording including reasons for acceptance or non-acceptance of description of the treatment concept in the indication section (i.e., symptomatic vs. disease modification-related wording).

- **Both regions:**
  ° **Recommendation 4:** In line with FDA and European Commission labeling guidelines, sponsors and agencies should ensure that the treatment concept is articulated in the label—to the extent that this can be supported by the available evidence—to facilitate communication of the product properties and conditions of use to patients and prescribers.
  ° **Recommendation 5:** The agencies are encouraged to consider including consultations with patients and prescribers in the final label approval process for discussions on how to best communicate the properties and treatment concept of new innovative medicines in a clear and comprehensible manner that reflects the available data.

### Conclusions

While in the EU there seems to be a higher degree of use of disease modification and related terminology outside the context of the product label (consistent referencing to disease modification in therapeutic drug development guidelines and lay language description suggesting a disease-modifying treatment concept in the Package Leaflet of MS drugs), we observed a clear trend from both the FDA and EMA toward a non-acceptance of disease modification-related terminology—or any claims specifying the treatment concept—in the label indication sections of drugs to treat MS.

Based on the learnings from the analysis of MS drug labels, we envision that future disease-modifying drugs for treatment of neurodegenerative disorders will be distinguished from symptomatic-only treatments by the description of the mechanism of action, clinical efficacy, and trial design as well as target patient population in the product label, at least until the state of the science allows for the demonstration of a convincing enduring effect on the disease course. This disposition is debatable: firstly, from a patient perspective, there is evidence suggesting that patients do not easily perceive information on benefit (67). Secondly, from a public health perspective, it could be challenging for prescribing physicians to readily retrieve information on how the drug works and what the effect will mean for the individual patient. Thirdly, the lack of description of the treatment concept in both the label and in the regulatory review documents may hamper discussions with payers. For example, it could be speculated that payers and HTA organizations might be more likely to accept some degree of extrapolation of the clinical benefit beyond the clinical trial duration as a basis for estimation of the cost-benefit analysis if there is evidence to support a disease-modifying effect as endorsed by the regulatory agencies. In contrast, if a disease-modifying effect is not distinguished from a symptomatic effect, this may lead to overly conservative modeling assumptions, which could eventually impact patient access. Finally, from an industry perspective, the apparent trend toward a higher degree of conformity in the label poses a challenge for selection of acceptable, fair, and balanced wording for description of the treatment concept in promotional materials.

While meaningful treatments that will effectively halt or change the course of these devastating disorders are still awaited, in terms of current and future labels, the aim should be to provide sufficient descriptive information to help healthcare professionals make the right prescribing decisions for the right patients.

## Author Contributions

AM designed the study, performed the analyses, interpreted the results, and wrote the manuscript. VJ performed the analyses, contributed to interpretation of the results, and critically reviewed and edited the manuscript. HV contributed to designing the study, performed the analyses, interpreted the results, and contributed to writing the manuscript.

### Conflict of Interest

All three authors are employees of H. Lundbeck A/S.

## Footnotes

^1^https://dailymed.nlm.nih.gov/dailymed/archives/index.cfm (accessed January 2019).
